# Stationary Population Dynamics Reveal a Structural Typology of Global Aging: A Binary Model Approach Across 195 Countries

**DOI:** 10.21203/rs.3.rs-6675831/v1

**Published:** 2025-06-11

**Authors:** James R. Carey, Arni S. R. Srinivasa Rao

**Affiliations:** 1 Department of Entomology, University of California, Davis 95616, USA; 2 Center for the Economic and Demography of Aging, University of California, Berkeley 94720, USA; 3 Medical College of Georgia, Augusta University, GA, USA; 4 Laboratory for Theory and Mathematical Modeling, Division of Infectious Diseases, Department of Mathematics, Augusta University, Georgia, GA, USA

## Abstract

This study presents a unified structural typology of global population aging using a novel Binary Population Pair framework. Grounded in the Stationary Population Identity, the approach pairs each observed national population with its stationary counterpart to quantify deviations across three core dimensions: age structure, population momentum, and longevity gains. Leveraging new comparative metrics, including the Lifespan Parity Ratio, Stationarity Gap, Terminal Dependency Ratio, Survival Offset, and Net Structural Aging, the study classifies 195 countries into a five-stage demographic succession model, from Youth Dominance to Age Dominance, with a transitional Youth-Age Crossover at the midpoint. Results reveal a broad convergence toward structural stationarity by 2100, as countries transition from youthful to increasingly gerontic profiles. A post-successional condition, the “Demographic Vortex,” is introduced to describe populations caught in a feedback loop of chronic low fertility and persistent structural aging. By integrating life lived and life left distributions, this framework captures aging as a directional, cumulative process. More fundamentally, it reconceptualizes aging not as a fixed trajectory toward senescence, but as a dynamic repositioning within an expanding and structurally shifting lifespan—transforming our understanding of what it means to age in the modern era.

## Introduction

Global aging is one of the defining challenges of the twenty-first century ^1^. Conventional indicators, such as the percentage of the population aged 65 and over, rely on fixed age thresholds and offer limited insight into the structural nature or progression of population aging^2–6^. To address this gap, we introduce a structural and temporal framework based on the Stationary Population Identity—a mathematical symmetry in which the age distribution of a stationary population precisely mirrors the distribution of remaining life years^7–9^. This underused principle allows us to pair each observed national population with its stationary counterpart, creating a binary model to compare age structure, demographic momentum, and survival offsets. From this foundation, we derive a five-stage typology of demographic succession that treats aging as a sequential, directional process. Unlike traditional methods that quantify aging through static measures or dependency ratios, our typology is grounded in life table–derived stationary population comparisons, offering a novel lens through which to assess both global patterns and national-level trajectories of demographic aging. Just as evolutionary phylogeny provides the structural basis for biological taxonomy, our demographic classification is rooted in fundamental population structure rather than arbitrary age-based thresholds. A novel endpoint, termed the Demographic Vortex, captures the fate of populations caught in a feedback loop of low fertility and persistent aging, culminating in what we call a Twilight Society, where traditional support systems and policy levers become unsustainable. This reframing shifts the concept of age from a numerical threshold to a relational and structural property, and aging from a static descriptor to a dynamic evolutionary process. Our framework thus offers a conceptual perspective and diagnostic tool for understanding national trajectories, informing global policy in the face of an aging century.

### Framework Overview

#### The Binary Population Pair Model

The Binary Population Pair Model consists of two sub-models: the Period Life Table Population Sub-Model, representing the theoretical stationary population, and the Observed Population Sub-Model, representing real-world populations. By pairing each observed population with its stationary counterpart, the model uses the stationary case as a scientific control to measure structural deviations. These deviations quantify the magnitude, direction, and offset of demographic aging, forming the basis for key structural metrics. Although conceptually central, the model’s application is foundational: it enables the creation of synthetic indicators that internalize stationary principles without requiring continuous direct comparison, allowing the framework to trace the dynamic, succession-like progression of population aging.

#### Pillars of Population Aging.

We believe that a comprehensive understanding of structural population aging rests on three interrelated demographic pillars: (1) Age structure, reflecting the distribution of individuals across life-course stages; (2) Population momentum, capturing the lagged effects of past fertility and mortality regimes on future age structures; and (3) Survival Offset, defined as the change in mean years remaining due to improvements in survival. Survival Offset functions as a mitigating force against structural aging by partially offsetting increases in average age. The interaction between rising age and survival gains gives rise to a derived metric, Net Structural Aging, which quantifies the portion of aging unmitigated by survival improvements. Together, these three pillars shape the underlying mechanics of population aging and inform widely used surface-level indicators such as the proportion aged 65 and older or the old-age dependency ratio. They define the core dimensions along which structural aging progresses and provide a coherent framework for analyzing and classifying demographic transitions over time.

**Fig. 1.**
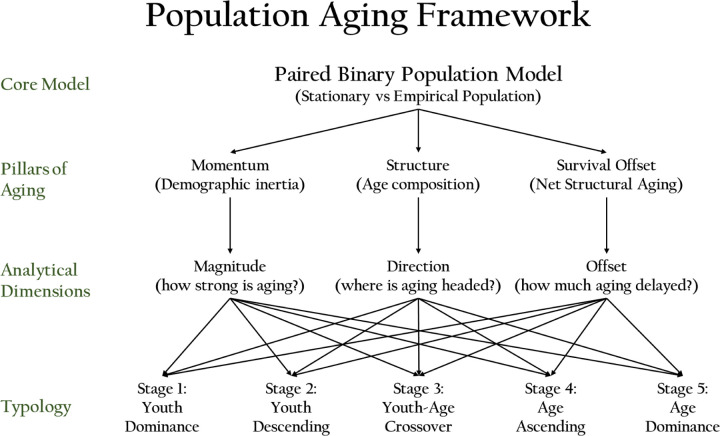
Conceptual framework of the Paired Binary Population Model and demographic aging typology. This schematic illustrates the structure of the Paired Binary Population Model, which compares each empirical population with its stationary counterpart to derive a multidimensional understanding of population aging. Lines connecting the analytical dimensions to the typology stages represent the composite basis of classification, reflecting the structural, directional, and temporal dynamics of aging as populations move from youth-heavy to age-dominant configurations

Figure 1 illustrates these three demographic pillars—age structure, momentum, and survival offset—and shows how they operate jointly to define the core dynamics of population aging. Together, they determine the magnitude, direction, and offset of demographic change, shaping both the progression of structural aging and the typological stage of a given population. This schematic serves as the conceptual backbone of our framework, grounding the succession model in clearly delineated dimensions.

Building on this three-pillar foundation, population aging unfolds through succession-like structural transformations. Demographic transitions follow a broadly sequential pattern, with populations shifting from youth-dominated to older age structures in a predictable order ^10–12^. The process is directional: as fertility declines and survival improves, populations move toward higher median ages, larger elderly shares, and smaller youth cohorts, with few instances of reversal ^2,13^. It is also largely irreversible; absent dramatic fertility rebounds or substantial youth-driven immigration, structural aging continues even with policy interventions ^3,14^. Thus, populations age through sequential, directional, and persistent transformations ^15,16^—patterns that stationary population comparisons help to formalize and classify.

### Visualization of Binary Population Pair Concept

**Figs. 2a,b.**
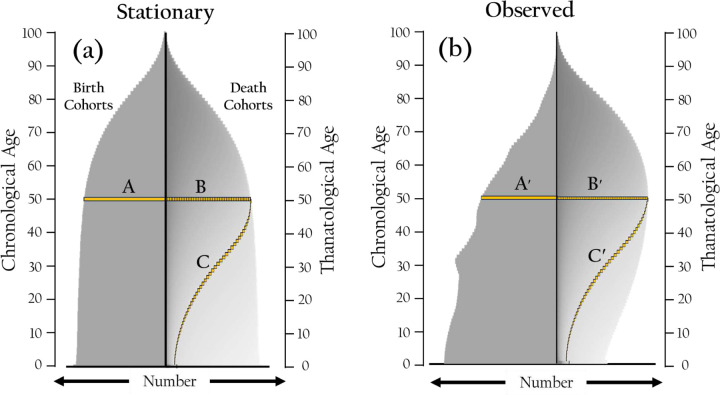
Distribution of birth and death cohorts ordered by chronological and thanatological ages. Data plotted are for the 2020 observed world population and its hypothetical stationary counterpart. Each figure features a birth-death cohort graph with two panels: the left shows the distribution of individuals by age as horizontal bar graphs, and the right shows projected deaths over time as stacked horizonal bar graphs with each stack in the bar containing the number of deaths in a given birth cohort at thanatological age x. In Fig. 2a (Stationary Population), the distributions are symmetrical, making Bar A, stacked Bar B, and the total in Diagonal Band C equal at approximately 95 million (Band C shows the time distribution of the 95 million deaths in the 50 year-old cohort). These reflect a symmetrical age structure where birth and death cohort distributions match. In Fig. 2b (Observed Population), Bar A′ (approximately 92 million) at age 50 represents individuals living at that age. Bar B′ (approximately 110 million) is a stacked bar representing individuals with at least 50 years remaining. Diagonal Band C′ (approximately 92 million) extends from Bar A to age 100, representing projected deaths for the 50-year-olds.

Figs. 2a and 2b use birth-death cohort graphs to visualize population data and concepts. These graphs resemble traditional population pyramids. In the left panels, they depict chronological age distributions, while the right-hand panels show the distribution of death cohorts rather than birth cohorts. Both stationary (1a) and observed (1b) cases are shown for comparison. The symmetry of the left and right panels in Fig. 1a allows for visual comparison with the actual world population in 2020.

Figs. 2a and 2b illustrate the distribution of birth and death cohorts, ordered from bottom to top by increasing chronological and thanatological ages. Fig. 2a shows the hypothetical stationary population, while Fig. 2b depicts the observed world population in 2020. Each figure contains two birth-death cohort graphs: the left panels show the distribution of individuals currently living by age, and the right panels display the projected distribution of deaths over time. In Fig. 2b, the left panel features a broad base, reflecting many young individuals, which is characteristic of a growing population. Bar A, located at age 50 in the left panel, represents the number of individuals currently living at this age in the 2020 world population (n = 92,581). The right panel shows a skew toward older ages, indicating younger individuals are expected to live longer. Bar B, a stacked bar in the right panel, corresponds to individuals from each cohort with at least 50 years remaining (n = 109,867). Diagonal Band C extends from Bar A in the left panel to age 100 in the right panel, representing the projected distribution of deaths for the 50-year-old cohort (n = 92,581). In contrast, Fig. 2a depicts a hypothetical stationary population with a symmetrical age distribution, reflecting balanced birth and death rates. The two gold-shaded bars and the diagonal band correspond to those in Fig. 2b, but in this stationary case, the values for both bars and the diagonal band are equal, indicating a stable population distribution (n = 94,919).”

**Fig. 3.**
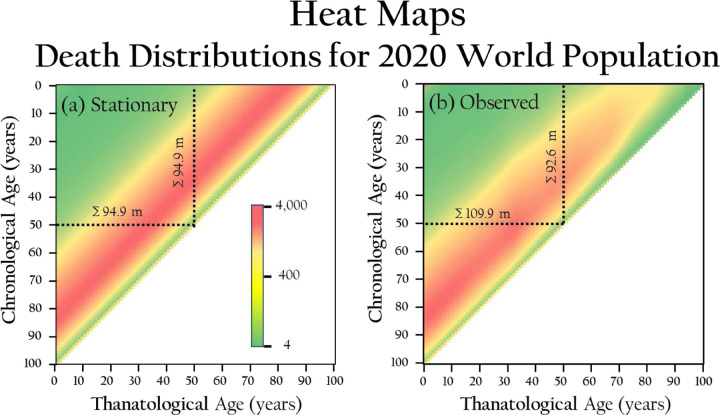
Heat maps of upper triangular matrices for the standing 2020 world population. Each graphic displays a 101 × 101 square matrix. In the first (left-most) column, the number of deaths by age are color-coded in cohorts aged 0 to 100 (top to bottom) at time 0. Deaths from birth cohorts are tracked across each row from left to right as the cohort ages until extinction at age 100 years. (a) Stationary Population: This map displays the distribution of remaining lives using data from the 2020 period life table for the world. In a stationary population, the sums of rows and columns are equal (e.g., sum of 50 year row equals sum of 50 year column), and all elements along each anti-diagonal are identical (i.e., colored “bands”). (b) Observed Population: This map shows the distribution of deaths based on calculations from the period life table and estimated cohort sizes. In this non-stationary population, neither the row and column sums nor the anti-diagonal elements are equal (e.g., sum of 50 year row and sum of 50 year column are not equal).

Figs. 3a and 3b show heat maps ^17,18^ of the distribution of remaining lives within population matrices for the 2020 world population, depicting stationary and observed cases, respectively. In each figure, the leftmost column shows the number of deaths by age group, ordered from youngest to oldest, for 2020 to 2021. Each subsequent column advances the age of each cohort by one year until reaching age 100, at which point they become extinct. In the stationary case (Fig. 3a), the sums of rows and columns for age 50 in 2020 and 50 years later are equal, illustrating the stationary population identity. This means that the number of individuals aged 50 equals those who die 50 years later, as the sequence of deaths over 50 years matches the sequence of 50-year-olds, 51-year-olds, etc., in the life table. Fig. 3b shows that the sums of the rows represent the number of individuals in each original age cohort from 2020. For example, 92.6 million deaths are projected among those who were 50 years old in 2020 from 2020 through 2070, as there were 92.6 million individuals in that cohort. The sum of each column represents the total number of deaths each year within the 100-year interval from 2020 to 2120. For instance, there will be 109.9 million deaths among the 50 non-extinct cohorts in 2070, 50 years after 2020.

### Global Population Aging Metrics

Table 1 illustrates the key population aging metrics using the observed 2020 world population and its hypothetical stationary counterpart, based on birth and death cohort distributions shown in Figs. 1a and 1b. This initial computation serves to introduce and contextualize the metrics, which are subsequently applied to 195 individual countries later in the paper. The comparison highlights the structural contrasts between real-world demographic patterns and the stationary benchmark defined by the Stationary Population Identity (SPI).

Conventional indicators—life expectancy at birth, old-age dependency ratio (OADR), and percent of the population aged 65 and older (%P_65_)— illustrate clear discrepancies. Although life expectancy at birth (e(0)) is identical in both populations at 72.0 years, the observed population is significantly younger, with an OADR of 14.3 compared to 29.0 and only 9.3% aged 65+ versus 18.0% in the stationary model.

The mean age in the observed population is 32.7, compared to 39.0 in the stationary case, while mean years remaining is higher in the observed case (44.2 vs. 39.0). This imbalance yields a Lifespan Parity Ratio (LPR) of 1.35 in the observed population, indicating a surplus of life left over life lived, compared to the SPI-defined value of 1.00 in the stationary population.

The Stationarity Gap (SG)—a measure of dissimilarity between actual and stationary age structures—was 0.2044, confirming significant age structural departure. The Terminal Dependency Ratio (TDR), which reflects the ratio of those within five years of death to the working-age population, was lower in the observed population (0.0716) than in the stationary model (0.1082), underscoring the demographic momentum still shaping global aging.

The Survival Offset (SO) for the world population was 1.6 years, and the Net Structural Aging (NSA) was 5.2 years, indicating that the total increase in mean age since 1970 was 6.8 years. This means that, absent gains in life expectancy, the average age of the world population would have increased by the full 6.8 years purely due to structural changes, namely, a recentering of the population around older ages. However, longevity improvements meant that the average individual in 2020 had 1.6 more years of life remaining than in 1970, which partially counteracted the demographic shift. Thus, SO reflects the added distance from death due to survival gains, while NSA captures the remaining structural aging burden; together, they explain how populations shift not just in age, but in their position relative to the endpoint of life. More detailed explanations of SO and NSA are given in Methods.

### Life Lived and Left: Comparison of World Regions

This framework builds on the mathematical foundations of the Stationary Population Identity, which establishes a symmetry between life lived and life left in stationary populations. The formulation and formal proof of SPI, its generalizations, and its broader implications for demographic analysis have been developed in earlier work by Carey and Rao and their collaborators. These include the original mathematical generalization and proof of the stationary population identity (Carey’s Equality) and related theorems on stationary and non-stationary populations ^19^, extensions to three properties of stationary populations and their knotting with non-stationary systems ^20^, and a comprehensive synthesis of the identity’s discovery, theoretical underpinnings, and applications across demographic contexts ^21^.

**Fig. 4a-f.**
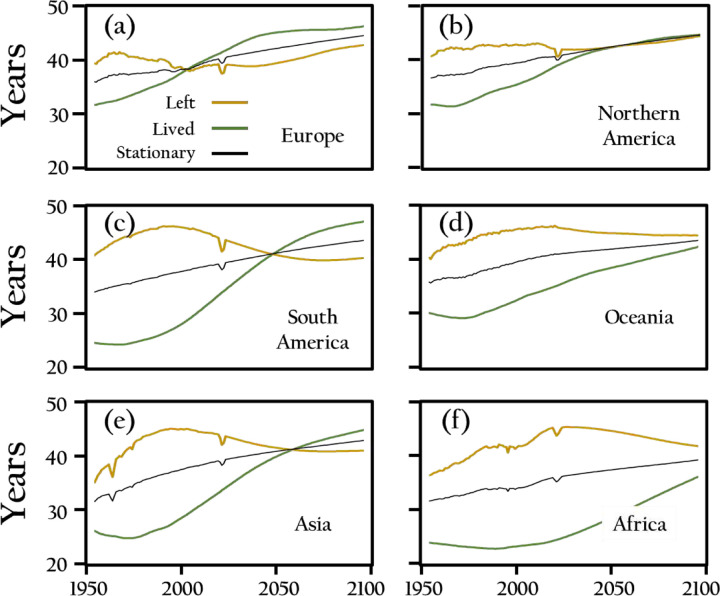
Trajectories for years lived, years remaining, and life table stationary age across six world regions from 1950 to 2100. Note the convergence of years lived and years remaining in all regions, including crossovers in Europe, South America, and Asia. Additionally, observe the intermediate trajectory of life table stationary ages, as determined from the period life tables for each year.

Figs. 4a–f show 150-year trends for lived years, remaining years, and stationary ages across six world regions. The subgraphs track changes in mean age (life lived) and mean years remaining (life left) from 1950 to 2100 in Africa, Asia, Europe, Latin America and the Caribbean, Northern America, and Oceania. The yellow line indicates the mean age, while the green line represents remaining years, with their intersection marking the stationary population age. In Africa, both metrics start low and rise gradually, with convergence around 2060. By 2100, the gap narrows but remains, reflecting a younger demographic despite improved life expectancy. Asia follows a steady increase, with convergence by 2040 and a stable, aging population by 2100. Europe shows a higher starting mean age, with an earlier crossover around 1980 and convergence by 2100. Latin America and the Caribbean, and Northern America follow similar patterns, with crossover around 2040–2060 and significant convergence by 2100. Oceania mirrors these trends, with convergence by 2100. Across regions, the crossover signals a shift toward older populations, occurring earlier in more developed regions like Europe and Northern America. By 2100, most regions approach a (quasi) stable age distribution, narrowing the gap between mean age and remaining years, emphasizing the global transition toward older populations.

### Historical and Future Trends

**Fig. 5a-d.**
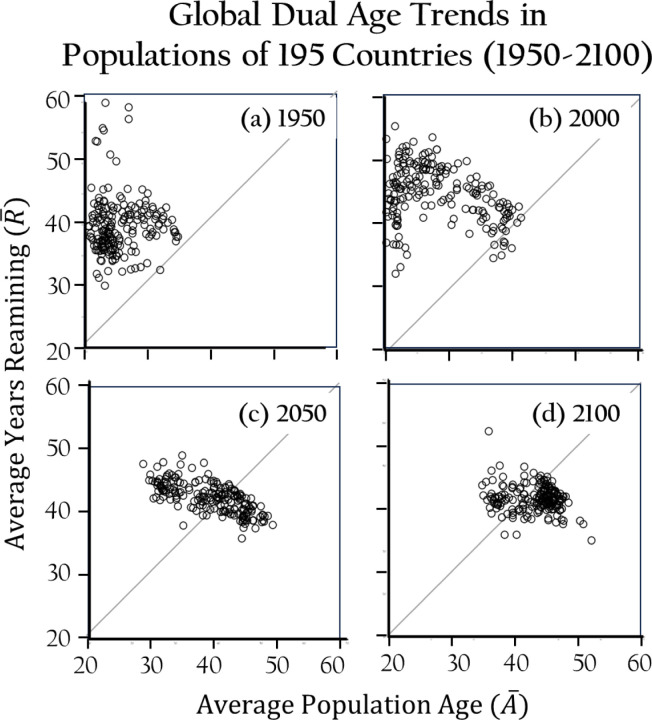
Plots of average years lived versus average number of years remaining. Plots incldue populations of 195 countries in 1950, 2000, 2050 and 2100. The number and percent of countries whose average population age was greater than the average years remaining was 2 (1%), 9 (4.5%), 73 (36.5%) and 136 (68%) for 1950, 2000, 2050 and 2100, respectively.

**Fig. 6.**
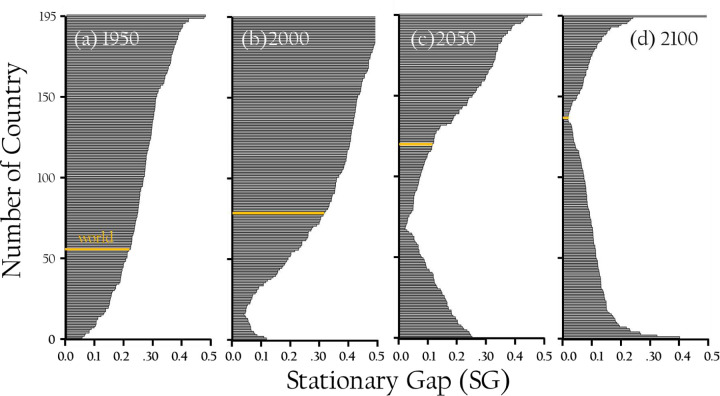
Horizonal bar graph distributions of Stationary Gap in 195 Countries, 1950–2100. These comparing the mean ages of the observed populations with their stationary complements, DIAge, for 195countries and the world population as a whole (gold-shaded bar) in four years from 1950 through 2100. The narrowest portion (shortest bars) of the distributions coincide with the change-over from postive population growth (above) to negative (below).

Figs. 5a–d illustrate the evolving relationship between average age and remaining years for populations across 195 countries from 1950 to 2100. In these plots, data points above the diagonal indicate younger populations with longer life expectancies, while those below represent older populations with fewer remaining years. In 1950, most countries displayed slightly older populations with lower life expectancies, as the post-war baby boom had not yet rejuvenated global age structures. By 2000, variability increased, with countries like Japan and Italy exhibiting aging populations due to low fertility, while regions like Africa maintained younger demographics. By 2050, populations start to converge, showing more uniform aging processes as fertility declines and healthcare improves. By 2100, this trend continues, with most countries aligned toward uniformly aging populations, with less disparity between average age and remaining years. Figs. 6a–d trace the evolution of age-structure Stationarity Gaps (SG) across 195 countries and the global population in 1950, 2000, 2050, and 2100. The Stationarity Gap measures divergence from a stationary age distribution, with values near zero indicating close alignment and values approaching 0.5 reflecting major structural disparity. Over time, gaps decline markedly: in 2000, many countries had SG values above 0.4, but by 2100 most fall below 0.15, signaling widespread convergence toward stationary-like structures as demographic transitions progress.

Each graph shows a distinct pinch point, marking the crossover from positive to negative population growth relative to age structure. Countries above this point experience positive momentum, driven by youthful cohorts, while those below it align more closely with stationary profiles, consistent with aging and decline. A high SG reflects strong demographic momentum—positive if skewed young, negative if skewed old—while a low gap suggests minimal momentum, with age distributions balanced around replacement-level fertility and mortality. Thus, the Stationarity Gap serves as a structural indicator of both the distance from stationarity and the likely future trajectory of population growth or decline based on current demographic composition.

**Fig. 7.**
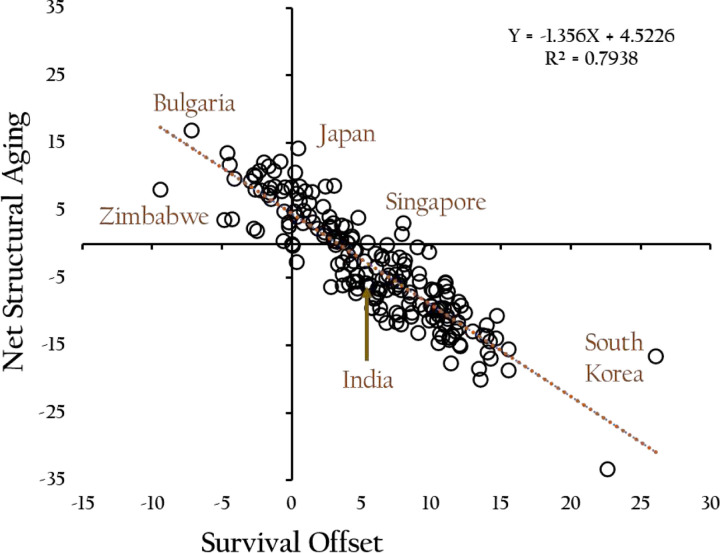
**Relationship between Survival Offset and Net Structural Aging across 195 countries, 2000.** Each point represents a country’s position based on the change in mean years remaining (Survival Offset, x-axis) and the difference between the increase in mean age and that offset (Net Structural Aging, y-axis). The downward-sloping linear trend reflects the inverse relationship between these two measures: populations with greater survival gains tend to experience less structural aging, and vice versa.

Fig. 7 illustrates the distribution of 195 countries by Survival Offset (x-axis) and Net Structural Aging (y-axis) in 2000. The placement of countries across the upper left, upper right, and lower right quadrants reveals distinct structural aging dynamics shaped by the interplay between longevity gains and chronological aging. Countries in the upper left quadrant exhibit negative survival offsets and positive NSA values. In these cases, mean years remaining declined over the period, while mean age increased—indicating unmitigated structural aging. However, this outcome does not reflect deteriorating mortality conditions. Rather, it results from the disproportionate effect of age structure changes, particularly long-standing low fertility, which caused the population to grow older even as survival gains were minimal or modest. The survival improvements that did occur were simply too small relative to the magnitude of structural aging. Countries such as Zimbabwe and Bulgaria exemplify this pattern, where aging has proceeded rapidly despite only modest or plateaued increases in life expectancy. These cases reveal how populations can age structurally even in the presence of non-declining, but insufficient, survival progress.

In the upper right quadrant, both survival offset and NSA are positive. Here, countries experienced improvements in survival—reflected in increased mean years remaining—but these gains were not sufficient to counterbalance the rise in mean age. Structural aging proceeded, albeit partially mitigated. Countries such as Japan exemplify this trajectory: although individuals are living longer, population aging continues due to persistent low fertility and a high proportion of older individuals.

The lower right quadrant contains countries with positive survival offsets and negative NSA values. In these populations, survival gains outpaced increases in mean age, resulting in structural rejuvenation. Though chronologically older, these populations became structurally younger relative to their lifespan. This reflects a demographic rebalancing, in which life left grew faster than life lived. South Korea and Singapore are prominent examples—countries that, through substantial gains in survival, have delayed or even reversed the structural aging process despite rising average ages.

These quadrant-based differences underscore the limitations of using chronological indicators alone to assess aging. The interaction between survival gains and structural change—captured by the Survival Offset and NSA—reveals a more nuanced and often counterintuitive demographic reality.

### Towards a Population Aging Typology

Building on the Binary Paired Population Model and the structural metrics introduced earlier, this section advances a typological framework to classify population aging systematically. Rather than applying arbitrary thresholds or descriptive indices, the typology draws on intrinsic contrasts between observed and stationary populations. We introduce the Stage Successional Aging Model, demonstrate its use across global population patterns from 1950 to 2100, and frame population succession as a form of demographic phylogeny. Although the model’s metrics—such as the Lifespan Parity Ratio and its subsidiary components—are derived solely from the observed population, they reflect the underlying binary logic of the stationary–observed comparison. The disaggregation of person-years into life lived and life left preserves the core symmetry of the stationary reference, maintaining conceptual alignment with the Binary Paired framework throughout.

The five-stage successional aging model offers a dynamic framework for understanding how populations evolve structurally over time. Each stage reflects a distinct configuration of demographic conditions, shaped by the interplay of age structure, demographic momentum, and the impact of longevity gains—captured through the Lifespan Parity Ratio (LPR) and its subsidiary metrics (Table 2).

#### Stage 1--Youth Dominance.

These populations are characterized by high fertility, low mortality, and a structurally young age distribution. Life left far exceeds life lived, as reflected in a high Lifespan Parity Ratio (LPR) of 1.93, indicating a large demographic reservoir of future person-years. The Stationary Gap (SG) is substantial (0.43), reflecting strong momentum driven by past fertility. The Terminal Dependency Ratio (TDR) is relatively low (0.10), and Survival Offset is high (8.55), signaling early-stage improvements in survival.

#### Stage 2—Youth Descent.

In this early transitional stage, fertility begins to decline and life expectancy rises. The population’s base narrows, and the age distribution begins to shift. The LPR declines to 1.43, indicating a measurable narrowing of the gap between life left and life lived. The SG decreases to 0.2399, signaling reduced demographic inertia, while TDR remains moderate (0.0987). LG turns slightly negative (−0.10), suggesting that while survival is improving, it is no longer offsetting structural aging. Aging is now emerging—present in the system but not yet dominant.

#### Stage 3—Youth-Age Crossover.

This stage represents not only the structural midpoint in the population aging process, but the conceptual anchor of the entire PAST model, reflecting the point of demographic stationarity where life lived and life left are equal. Fertility and mortality have flattened, and the population begins to approximate demographic stationarity. The LPR is near 1.0 (0.98), indicating balance between life lived and life left. The SG reaches its lowest point (0.0559), showing minimal deviation from stationary structure. TDR rises slightly to 0.1144, and LG becomes more clearly negative (−0.20), reflecting diminishing returns from longevity improvements. This stage serves as the system’s pivot point from which structural aging accelerates.

#### Stage 4—Age Ascent.

Aging becomes the dominant structural force. Fertility is well below replacement, and older cohorts exert increasing influence on the population structure and social systems. The LPR declines to 0.88, indicating that life lived now exceeds life left for the average individual. SG increases modestly to 0.1056, while TDR rises to 0.1490, highlighting a growing end-of-life burden. LG, now −0.09, is insufficient to offset structural aging.

#### Stage 5—Age Dominance.

This stage marks the terminal phase of the demographic succession. Fertility is chronically low, mortality improvements have slowed, and the age structure is dominated by older adults. The LPR declines further to 0.81, the lowest of all stages. SG increases again to 0.1673, and TDR reaches its highest level at 0.1891, signaling substantial structural and health-related dependency. LG, at −0.21, confirms that survival improvements are no longer delaying aging pressures. This stage reflects a population in structural decline, defined by longevity, high dependency, and limited internal renewal.

### The Demographic Vortex and the Twilight Society

While Stage 5 represents the most advanced form of structural aging within the typology, it does not mark the end of demographic evolution. Without renewal—through sustained fertility recovery, immigration, or generational rebalancing—societies may enter what can be described as a Demographic Vortex: a self-reinforcing spiral in which shrinking cohorts, rising old-age dependency, and weakening policy efficacy accelerate the transition toward demographic irreversibility. The vortex is not a stage, but a dynamic descent, defined by compounding structural inertia and diminishing prospects for recovery. Ultra-low fertility is the primary catalyst for potential entry into the Demographic Vortex. Goldin’s “quiet revolution” shows how expanded education and career opportunities delayed marriage and childbearing, trends supported by access to contraception ^22,23^ and rising opportunity costs. These shifts are closely aligned with the Second Demographic Transition framework, which emphasizes changes in values and social norms, particularly the rise of individual autonomy, self-actualization, and shifting family expectations, as the underlying force driving persistent below-replacement fertility across much of the developed and developing world. ^24^. These individual-level trends, including the growing social acceptance of voluntary childlessness, have been extensively studied in both theoretical and cross-national contexts^25–27^. Reinforced by global family-planning initiatives, these changes have driven fertility rates below replacement in most countries, setting the stage for sharp population declines, with many projected to lose over 50% of their populations by 2100 ^11^. Nowhere is this trajectory more visible than in South Korea, where fertility fell from 6.33 in 1960 to 0.78 in 2022 despite extensive pronatalist efforts^14^.

Emerging from this vortex is not simply a more aged population, but potentially a transformed social order—a Twilight Society. This is not a dystopia, but a civilization shaped by the dominance of age and the fading of generational succession. In a Twilight Society, forward-looking institutions give way to those oriented toward care, memory, and managed decline. Political influence consolidates in the hands of older cohorts; cultural narratives become retrospective; investment in youth contracts. It is a society that has not collapsed but has turned inward. This post-Stage 5 condition underscores the importance of understanding population aging not just as a structural process, but as a successional transformation of the societal life cycle.

### Historical Patterns: A Successional Stage Analysis

The distribution of countries across the five successional stages of population aging—from Youth Dominance (Stage 1) to Age Dominance (Stage 5)—reveals a compelling narrative of global demographic transformation over the 150 years from 1950 to 2100 (Table 3). These patterns underscore not only the temporal directionality of aging, but also the structural and geographic diffusion of demographic change across the globe.

#### 1950—World at the Dawn of Aging.

The global demographic landscape was overwhelmingly youthful. Stage 1 (Youth Dominance) included countries such as Brazil, the Philippines, Turkey, Ghana, and Peru, reflecting high fertility, low median age, and rapidly expanding population structures. With 33 countries classified in this stage, populations exhibited LPR values well above 1.75, indicating that life left overwhelmingly exceeded life lived.

The largest share of countries (159) resided in Stage 2 (Youth Descent), including India, Iran, Thailand, South Africa, and Indonesia. These populations had begun their demographic transition, marked by declining fertility and rising life expectancy, though the structural consequences of aging were still modest. Only five countries—including Austria, Belgium, Luxembourg, the United Kingdom, and Mayotte—had entered Stage 3 (Youth-Age Crossover). These were the earliest examples of populations reaching near-parity between years lived and years left (LPR ≈ 1.0), suggesting a stable, mature age structure with minimal demographic momentum. No countries were in Stage 4 (Age Ascent). South Korea stood alone in Stage 5 (Age Dominance), an anomaly reflecting a unique structural profile likely due to war-induced demographic distortion.

#### 2000—Towards a Global Inflection Point.

By 2000, significant global demographic shifts had occurred. Stage 1 populations remained sizable (100 countries) but are now concentrated in low-income or conflict-affected regions such as Afghanistan, Angola, Nigeria, Tanzania, and Pakistan. Stage 2 include middle-income emerging economies like Brazil, India, Indonesia, South Korea, and Turkey—countries where aging had become demographically visible but had not yet restructured societal institutions. The number of countries in this stage declined to 72, indicating movement toward structural consolidation. Stage 3 grew to 26 countries, now including Germany, Italy, Spain, the UK, and Sweden. These populations exemplified balanced age structures with low momentum and maturing support systems. Bulgaria, with its combination of low fertility, declining population size, and increasing old-age burden, was the sole country classified in Stage 4. Still, no countries had yet crossed into Stage 5, though the thresholds were approaching for some deeply aging societies.

#### 2050—The Ascent of Age Dominant Societies.

The landscape by 2050 marks a demographic turning point. Stage 1 has shrunk to just 12 countries, predominantly in sub-Saharan Africa (e.g., Angola, Burkina Faso, Mali, Niger, Somalia), where fertility remains high and structural aging has yet to take hold. Stage 2 is still populous (86 countries), including India, Pakistan, Nigeria, Kenya, and Egypt—nations with large cohorts still transitioning from high to moderate fertility and longevity improvement. These populations are structurally youthful but aging fast. Stage 3 rises significantly to 56 countries, indicating that structural maturity is no longer confined to high-income nations. The United States, Brazil, Turkey, Indonesia, and Australia now exemplify stable aging with declining demographic momentum. In Stage 4, now with 10 countries, we see nations like China, the Netherlands, Portugal, Czechia, and Finland where the aging process has become the dominant structural force. These countries have LPRs well below 1.0 and rising dependency burdens. Stage 5 emerges clearly for the first time, with 35 countries— including Japan, Italy, Germany, South Korea, and Spain. These populations are demographically inverted, with more years lived than left on average, minimal fertility recovery, and long-term structural contraction.

#### 2100—A Deeply Aged World.

By 2100, the global demographic distribution will be fundamentally reshaped. No country remains in Stage 1 indicating that structural youthfulness has disappeared. Stage 2 includes 42 countries, among them Nigeria, Kenya, Iraq, Senegal, and Cameroon. These represent the last wave of incipient aging societies, many of which are only now entering demographic transition due to late-stage fertility decline and rising survival. Stage 3 becomes the modal category with 77 countries, including large, regionally diverse nations such as the USA, India, Brazil, France, and Iran. These countries have reached structural parity and stability, with moderate aging and low momentum. Stage 4, with 40 countries, now includes a global mix—Portugal, China, Estonia, Mexico, and Latvia—nations in which aging exerts profound economic and social pressure despite robust institutions. Finally, Stage 5 encompasses 40 countries, including Japan, Italy, South Korea, Thailand, and Germany. These are societies with permanently inverted age structures, ongoing natural population decline, and increasingly complex demands for eldercare and economic adaptation.

### Contrasting Conventional and Succession-Based Classifications

While widely used, conventional aging metrics such as the percentage of the population aged 65+ and the old-age dependency ratio (OADR) often fail to capture the full demographic complexity of aging societies. Several countries with similar values for these indicators occupy different stages in our five-stage succession typology, revealing structural and temporal contrasts that conventional metrics cannot detect.

For instance, in 2050, France and Hungary have nearly identical fractions over age 64 (27.8% vs. 28.0%) and similar OADRs (0.49 vs. 0.48), yet France is classified as Stage 3, reflecting transitional aging, while Hungary is in Stage 5, indicating structural aging saturation. The key difference lies in France’s modest negative survival offset (−0.08) and high structural aging (NSA = 7.18), versus Hungary’s positive survival offset (+0.93) and a lower NSA of 6.53—highlighting France’s demographic momentum and Hungary’s entrenched structure.

Similarly, Ireland (2050) and Serbia (2050) have nearly equal %65+ populations (~26–27%) and OADR values (~0.44–0.46), but Ireland is in Stage 3 while Serbia is in Stage 5. Ireland’s strongly negative survival offset (−1.09) and NSA of 11.3 indicate an aging process still in motion, whereas Serbia, despite similar surface values, has already structurally stabilized with lower momentum.

At the opposite end, countries like Senegal and Congo (DRC) in 2050 both show extremely low old-age shares (<6%) and similar OADRs, yet they remain in Stage 1, reflecting youthful population structures and strong survival gains. However, Uganda and Congo (not DRC) are in Stage 2, despite comparable conventional metrics, due to greater structural aging (NSA = 3.34 and 1.66 respectively), signaling early demographic shifts not captured by thresholds alone.

These comparisons underscore that populations with similar chronological aging profiles may be structurally and temporally divergent. The succession model captures this by embedding the population within an evolutionary framework of demographic change, informed not only by age structure but also by survival trajectories and stationary comparisons.

### Path to Senescent Twilight States: East Asia and Southern Europe

A growing number of countries face the risk of entering a demographic vortex and evolving into senescent twilight states—nations maintaining sovereignty but hollowed demographically by extreme aging and population decline. In East Asia, South Korea, Japan, and Taiwan are at the forefront. Fertility rates have collapsed to historic lows, rigid social structures inhibit adaptation, and immigration remains politically constrained. Without profound societal shifts, these nations may retain their political identities even as their demographic foundations erode. In Southern Europe, Italy, Spain, Greece, and Portugal confront a parallel crisis. Persistent low fertility, chronic economic stagnation, and mass youth emigration have already led to significant rural abandonment and aging cities. While some regions struggle to attract immigrants, the scale of decline often outpaces policy responses. Together, these East Asian and Southern European cases illustrate how different societal systems, despite geographic and cultural distance, may converge toward a shared future: sovereign but aged, persistent but hollow—senescent twilight states awaiting their demographic twilight.

## DISCUSSION

This study introduces a unified scientific model of population aging that transcends traditional descriptive metrics. To contextualize our approach, we first examine historical efforts to characterize population aging, highlighting their contributions and limitations. Building upon this foundation, we delineate the theoretical underpinnings of our framework, emphasizing its departure from conventional methodologies. Finally, we explore how our model complements existing policy-oriented indicators, offering a more nuanced understanding of demographic aging.

### Population Aging: Historical Background.

Efforts to characterize population aging have traditionally relied on simple, descriptive indicators. Measures like the percentage aged 65 and older and the old-age dependency ratio (OADR) remain widely used, offering interpretable but surface-level snapshots. While useful for broad comparisons, these metrics overlook deeper demographic processes such as fertility decline, survival gains, and structural momentum.

Recognizing these limitations, demographers introduced more dynamic perspectives. Preston et al. ^28^ embedded aging within broader demographic forces— mortality, fertility, and migration—while Swanson, Tedrow, and colleagues ^29,30^ highlighted demographic algebraic identities that reveal the mechanics of population change. Relatedly, Kim and Schoen ^31^ and Espenshade et al. ^32^ tied momentum effects to aging trajectories, showing how fertility reductions accelerate structural aging. Collectively, these studies reframed aging as an emergent outcome of demographic transitions rather than a static endpoint.

Other research reconceptualized the meaning of age itself. Riffe and colleagues ^33–35^ introduced thanatological age—time until death—as a key structural dimension, symmetric with chronological age in stationary populations. Sanderson and Scherbov ^5,13,36,37^ advanced the concept of prospective age, redefining age by remaining life expectancy. Wrigley-Field and Feehan ^38^ proposed the “average lifespan of the living” as an alternative to conventional life expectancy.

At the composite level, indices such as the Global AgeWatch Index ^6^ and the Aging Society Index (Chen et al. 2018) benchmarked national outcomes across multiple domains but remained fundamentally descriptive. Additional refinements include Guillot’s ^39^ mortality momentum, Balachandran’s ^40^ Comparative Prospective Old Age Threshold, and Chang et al.’s ^41^ advocacy for health-adjusted measures like DALYs.

Despite these advances, existing typologies remain fragmented and externally imposed, lacking a structural foundation that captures the sequential dynamics of demographic aging. This gap motivates the present study, which introduces a binary stationary model to classify aging stages based on internal demographic logic. Recent global analyses by Ma et al. ^42^ underscore the near-universal relevance of this structural perspective.

### A Unified Scientific Model.

Unlike virtually all of the conventional approaches described above to classifying aging populations which primarily serve as descriptive metrics, our framework represents a unified scientific model. First, our model is anchored in deep mathematical theory, particularly the stationary population identity^7,9,19^. This identity, a mathematical truth, equates the distributions of life lived and life left in a stationary population, providing a rigorous basis for deriving metrics that diagnose aging as a dynamic, structural condition rather than merely a descriptive trend. Second, our approach synthesizes concepts from the social sciences, formal sciences, and natural sciences. It combines demographic analysis with mathematical modeling and biological principles, creating a comprehensive framework that transcends traditional disciplinary boundaries and offers a holistic understanding of population aging. Third, our framework is informed by the principles of ecological succession^43^ and evolutionary phylogeny^44^ to classify aging populations into empirically grounded stages. Just as phylogenetic systematics in biology organizes species based on evolutionary relationships and shared derived characteristics, our approach categorizes populations not by their descriptive traits, but by their positions within a structural lineage of demographic evolution ^16^. Fourth, despite the vast literature on population aging, there is a striking absence of discussion around its end process and terminal condition. Here we fill that void by introducing the concepts of the *Demographic Vortex* as the mechanism initiating entry into, and the *Twilight Society* as the absorbing state of irreversible aging. This framework underscores the potential for long-term demographic entrapment beyond current projection horizons, highlighting the necessity of understanding and addressing the structural dynamics leading to such irreversible conditions ^20^.

Furthermore, the metrics developed and utilized within our typology—namely, the lifespan parity ratio, terminal dependency ratio, stationary gap, and longevity gains—are intrinsically designed to capture the dual aspects of demographic structure: chronological age and remaining years of life. By incorporating both dimensions, our framework transcends traditional age-based metrics, offering a more comprehensive and structurally grounded understanding of population aging dynamics.

### Complementarity with Conventional Metrics.

A key strength of the successional stage model lies in its ability to complement, rather than replace, existing policy-oriented metrics that assess the challenges of aging societies. Indicators such as the percentage aged 65 and over, the Old-Age Dependency Ratio, and the Global Aging Society Index focus primarily on societal burdens or adaptive capacity ^45^. While essential for policy planning, these measures operate mainly at the level of societal response rather than intrinsic demographic structure.

The successional stage model adds a deeper dimension by classifying populations according to their internal structural trajectory, grounded in demographic fundamentals such as age distribution shape, stationary gaps, lifespan parity, and terminal dependency. Whereas %≥65 and OADR provide surface indicators and the Global Aging Society Index assesses adaptive readiness, the succession framework situates populations along a universal demographic continuum based on underlying structural dynamics ^46^.

This complementarity enhances both scientific understanding and policy relevance. It distinguishes where a population stands within the aging process, independent of socioeconomic context, while conventional metrics evaluate how well it is adapting. Together, these perspectives offer a more nuanced and actionable understanding of the timing, magnitude, and policy implications of global demographic aging. As Bloom et al. 2 emphasize, integrating structural demographic analysis with adaptive policy frameworks is critical for anticipating and responding effectively to the profound societal transformations driven by global aging.

### Dance of the Ages: Where Chronological and Thanatological Time Converge.

Grounded in the stationary population identity, which equates years lived with years left, our framework reconceives aging not as a linear progression, but as a dynamic balance along the human lifespan. Population aging emerges as a “dance” between two opposing forces. On one side is the chronological pull toward death, as structural aging shifts the population’s age distribution toward older cohorts, drawing the center closer to the end of life. On the other is the thanatological push away from death, as rising life expectancy extends the average years remaining, distancing the population from its mortality horizon. The result is a repositioning of the demographic center, not fixed in age but continually balanced between the weight of accumulated age and the expanding span of life left. This interplay determines whether a population moves closer to or further from death, not just in years lived, but in how it is structurally composed and temporally aligned within the human lifespan. In capturing this interplay, our framework redefines population aging as a dynamic repositioning within the lifespan, offering a deeper understanding of how aging populations evolve, shaped not simply by the passage of time, but by the shifting balance between years lived and years left.

## Methods

### The Stationary Population Identity.

Central to our framework is the Stationary Population Identity (SPI), an important demographic relationship discovered independently by Brouard ^47,48^ and Carey ^7–9^. In a closed stationary population with fixed vital rates, the proportion of individuals at age x equals the proportion with x years remaining. For example, if 2% of a stationary population are under age 3, then exactly 2% have no more than 3 years left to live.

Formally, if $\text{c}\left(\text{x}\right)$ denotes the fraction of individuals age x and $\text{g}\left(\text{x}\right)$ denotes the fraction with x years left to live, then:

(1) $$c\left(x\right)=g\left(x\right)$$

The age-weighted integrals of each side over all ages given as:

(2) $$\int_{0}^{\omega }xc\left(x\right)dx=\int_{0}^{\omega }xg\left(x\right)dx$$

gives the equality of the mean age of the population (left integral, denoted $\bar{\text{c}}$) and the mean number of years remaining for the average individual (right integral, denoted $\bar{\text{g}}$):

(3) $$\bar{c}=\bar{g}$$

Kim and Aron ^49^ independently formalized this same relationship, showing that in a stationary population, the average age is equal to the average remaining life expectancy.

### Measuring the Dimensions of Population Aging

#### Conventional Reference Metrics

Although the focus of our study is on metrics associated with the stationary population, the stationary identity and the stationary population matrix, we include computation of two conventional metrics for context and perspective. The first is the percent over 65, denoted %𝑃_65_, in a population given as

(4) $$\%P_{65=}\frac{P_{65+}}{P_{total}}$$

where $P_{65+}$ and $P_{total}$ denote population 65 and older and total population, respectively. The second conventional metric is the Old Age Dependency Ratio (OADR) which represents the ratio of the population aged 65 and older to the working-age population defined as those aged 15 to 64, denoted $P_{15-64}$:

(5) $$OADR=\frac{P_{65+}}{P_{15-64}}$$

#### Structural Metrics from the Binary Population Pair

The following metrics are uniquely derived from our binary population pair model. The Terminal Dependency Ratio (TDR) measures the burden of imminent mortality relative to the working-age population, based on projected deaths within five years. It uses actual population counts at each age weighted by age-specific mortality probabilities. Let

$$n\left(x,t\right)\;=\;\text{number}\;\text{of}\;\text{individuals}\;\text{aged}\;\text{x}\;\text{at}\;\text{time}\;\text{t}$$

$$q\left(x,t\right)\;=\;\text{probability}\;\text{of}\;\text{death}\;\text{age}\;\text{x}\;\text{at}\;\text{time}\;\text{t}$$

The expected number of deaths at age x over the next five years, assuming independent annual mortality, is approximated by:

(6) $$d_{5}\left(x,t\right)=n\left(x,t\right)\times (1-\left[1-\prod_{0}^{4}q\left(x+i,t+1\right)\right]$$

Then the terminal population $\text{T}\left(\text{t}\right)$ is the sum across all ages:

(7) $$T\left(t\right)=\sum_{0}^{\omega }d_{5}\left(x,t\right)$$

The number in the population aged 20 to 64, denoted $P_{20-64}$ is given as

(8) $$P_{20-64}=\sum_{20}^{64}n\left(x,t\right)$$

Thus, the Terminal Dependency Ratio (TDR) is calculated as:

(9) $$TDR=\frac{T\left(t\right)}{\sum_{20}^{64}n\left(x,t\right)}$$

A higher TDR indicates greater imminent mortality burden relative to the working-age population, while a lower TDR suggests reduced short-term dependency pressure. Unlike the Old-Age Dependency Ratio, which scales directly with the proportion aged 65 and older, the Terminal Dependency Ratio varies widely among countries with low elderly shares—revealing underlying mortality dynamics not captured by standard metrics. This divergence is illustrated in a two-panel comparative analysis in Appendix Fig. III-1.

The mean age in the observed population, ${\bar{A}}_{0}$, and the mean age in the stationary population, ${\bar{A}}_{s}$, representing the average age of individuals in each respective population, are calculated as follows:

(10) $${\bar{A}}_{0}=\frac{\sum_{x=0}^{\omega }xn_{o}\left(x,t\right)}{\sum_{x=0}^{\omega }n_{o}\left(x,t\right)}$$

(11) $${\bar{A}}_{s}=\frac{\sum_{x=0}^{\omega }xn_{s}\left(x,t\right)}{\sum_{x=0}^{\omega }n_{s}\left(x,t\right)}$$

where $n_{0}$ and $n_{s}$ refer to the number of individuals at age x and time t in the observed and stationary populations, respectively.

The mean years remaining, $\bar{R}\left(t\right)$ denoting the number of years the average individual in the population has left to live at time t is given as

(12)</ $${\bar{A}}_{R}=\frac{\sum_{x=0}^{\omega }l\left(x,t\right)e\left(x,t\right)}{\sum_{x=0}^{\omega }l\left(x,t\right)}$$

where $\text{l}\left(\text{x,t}\right)$ denotes the number of survivors to age x at time t, and $\text{e}\left(\text{x,t}\right)$ denotes the remaining life expectancy at age x and time t.

The Lifespan Parity Ratio, $\text{LPR}\left(\text{t}\right)$ compares the mean years remaining to the mean chronological age:

(13) $$LPR=\frac{\bar{R}\left(t\right)}{{\bar{A}}_{o}\left(t\right)}$$

An LPR greater than 1.0 signals a youthful population, an LPR of 1.0 indicates stationarity, and an LPR less than 1.0 reflects an aged population with more years lived than remaining.

#### Momentum-related Metrics

The Stationarity Gap (SG) quantifies the divergence between an observed population and its corresponding stationary population. Adapted from the classic Dissimilarity Index ^50,51^, it measures the unevenness between two distributions and ensures values between 0 (perfect similarity) and 1 (maximum divergence). In a perfectly stationary population, the number of individuals aged x would equal the number of individuals with x years remaining. Thus, deviations between these two distributions provide a direct measure of how far the observed population is from perfect stationarity.

We define the Stationarity Gap, denoted SG, as follows:

(14) $$SG=\frac{1}{2}\sum_{x=0}^{\omega }\left|\frac{n\left(x,t\right)}{N}-\frac{d\left(x,t\right)}{D}\right|$$

where: n(x,t) = number of individuals aged x at time t (age distribution), d(x,t) = number of individuals with x years remaining at time t (remaining years distribution), N = total number of individuals in the observed age distribution, D = total number of individuals in the observed remaining-years distribution, and ω = maximum age considered. A value of SG=0 indicates perfect stationarity, while values greater than 0 reflect demographic momentum—positive if the population is growing, negative if it is declining—that drives continued structural change over time.

#### Survival Offset and Net Structural Aging

To assess how improvements in survival mitigate the progression of structural population aging, we define two related metrics: Survival Offset (SO) and Net Structural Aging (NSA). These are based on the temporal relationship between changes in mean population age and mean remaining years.

Let:

$${\bar{A}}_{O}\left(t\right)=\;\text{mean}\;\text{age}\;\text{of}\;\text{the}\;\text{observed}\;\text{population}\;\text{at}\;\text{time}\;\text{t}$$

$${\bar{A}}_{R}\left(t\right)=\;\text{mean}\;\text{remaining}\;\text{years}\;\text{of}\;\text{the}\;\text{observed}\;\text{population}\;\text{at}\;\text{time}\;\text{t}$$

We compute changes over a specified time interval:

(15) $$\Delta {\bar{A}}_{O}={\bar{A}}_{O}\left(t\right)-{\bar{A}}_{O}\left(t-1\right)$$

(16 $$\Delta {\bar{A}}_{R}={\bar{A}}_{R}\left(t\right)-{\bar{A}}_{R}\left(t-1\right)$$

The Survival Offset (SO) is defined as the change in the mean years remaining:

(17) $$SO=\Delta {\bar{A}}_{R}$$

The Net Structural Aging (NSA) represents the portion of aging not mitigated by survival gains:

(18) $$NSA=\Delta {\bar{A}}_{O}-SO=\Delta {\bar{A}}_{O}-\Delta {\bar{A}}_{R}$$

Survival Offset (SO) measures how much the average position of individuals in a population has shifted relative to death over time. It reflects the net effect of two opposing demographic forces: (1) rising life expectancy, which extends the distance to death, and (2) structural population aging, which compresses that distance by recentering the population around older ages. A positive SO means the average person in the population now has more years remaining—that is, they are farther from death than 50 years ago, thanks to longevity gains outpacing structural aging. A negative SO indicates that the average person is now closer to death, not because survival has worsened, but because the population has grown older so substantially that structural aging has overtaken survival improvements. In this case, the age structure has “recentered” the population around later life, reducing average remaining years despite longer life spans. Net Structural Aging (NSA) represents the portion of chronological aging that has not been offset by gains in life expectancy. It is calculated as the difference between the change in mean chronological age and the Survival Offset. It measures the residual demographic aging—how much the population has moved structurally closer to death, even after accounting for improved survival. High NSA values indicate deep, unmitigated structural aging, while low NSA values suggest that longevity gains have cushioned much of the demographic shift. Together, SO and NSA decompose the process of aging into its temporal (life expectancy) and structural (age composition) components, showing not just that a population is aging, but how and why its position relative to death is changing over time.

### Data Sources

We used two complementary datasets from the United Nations for both sexes combined, covering the period from 1950 to 2100 ^52,53^. These datasets include: (1) the number of individuals in a population by age $\left(\text{x}\right)$ for each country by year $\left(\text{t}\right)$, denoted $\text{n}\left(\text{x,t}\right)$; and (2) the corresponding life table information, including age-specific cohort survival $\text{l}\left(\text{x,t}\right)$, mortality $\text{q}\left(\text{x,t}\right)$, and expectation of life $\text{e}\left(\text{x,t}\right)$. Methodology for estimating fertility, mortality and migration for the population projections are given in ^54^.

## Figures and Tables

**Table 1.** Population aging metrics for the observed 2020 world population (observed) and its hypothetical stationary case.

Metric	Observed	Stationary
**Conventional Metrics**		
Expectation of life at birth, e(0)	72.0	72.0
Old Age Dependency Ratio (OADR)	14.3	29.0
Percent 65 and older ($\%P_{65}$)	9.3	18.0
**Lived and Left**		
Mean Age (${\bar{A}}_{O}$; ${\bar{A}}_{S}$)	32.7	39.0
Mean Years Remaining (${\bar{A}}_{R}$)	44.2	39.0
**Keystone Metric**		
Lifespan Parity Ratio (𝐿𝑃𝑅)	1.35	1.00
**Subsidiary Metrics**		
Terminal Dependency Ratio (TDR)	0.0716	0.1082
Stationarity Gap (SG)	0.2044	0.0000
Survival Offset (SO)	1.6	3.6
Net Structural Aging (NSA)	5.2	0.0

**Table 2.** Successional stages of population aging and associated metrics. All metrics are derived from the joint analysis of life left and life lived distributions. Stage classification is based exclusively on the Lifespan Parity Ratio (LPR), the keystone metric generated from the binary distribution pairing.* Stage 3 (Youth-Age Crossover) in green font is the structurally quasi-stationary stage. Reported values represent medians, with interquartile ranges in parentheses (25th–75th percentiles).

	STAGE				
STAGE	Youth Dominance 1	Youth Descent 2	Youth-Age Crossover 3	Age Ascent 4	Age Dominance 5
**Lifespan Parity Ratio**	1.93 1.84–2.06	1.43 1.23–1.59	0.98 0.94–1.04	0.88 0.86–0.89	0.81 0.77–0.83
**Terminal Dependency Ratio**	0.10 0.07–0.15	0.10 0.08–0.16	0.11 0.10–0.13	0.15 0.14–0.16	0.19 0.18–0.14
**Stationary Gap**	0.43 0.39–0.47	0.24 0.15–0.30	0.06 0.04–0.07	0.11 0.01–0.12	0.17 0.14–0.20
**Survival Offset**	8.55 5.78–11.19	−0.67 −2.82–3.51	−1.51 −4.21–0.81	−.0.34 −2.36–2.26	−1.68 −3.85–1.50
**Net Structural Aging**	−8.97 −11.71-(−4.96)	7.56	9.78 4.86–15.12	7.38 1.29–11.14	10.68 4.54–15.60

* Stages are defined by the following LPR thresholds: Stage 1 (Youth Dominance): LPR > 1.75; Stage 2 (Youth Descent): 1.10 < LPR ≤ 1.75; Stage 3 (Youth-Age Crossover): 0.90 < LPR ≤ 1.10; Stage 4 (Age Ascent): 0.85 < LPR ≤ 0.90; Stage 5 (Age Dominance): LPR ≤ 0.85

**Table 3.** Countries by successional stage of population aging and year Each row lists five representative countries for each of the five demographic aging stages—Youth Dominance (Stage 1) through Age Dominance (Stage 5)—for the years 1950, 2000, 2050, and 2100. The number in parentheses indicates the total number of countries assigned to that stage in the given year, based on their Lifespan Parity Ratio (LPR).

	STAGE				
Year	Youth Dominance 1	Youth Descent 2	Youth-Age Crossover 3	Age Ascent 4	Age Dominance 5
1950	Brazil, Philippines, Turkey, Ghana, Peru (33)	India, Iran, Thailand, South Africa, Indonesia (159)	Austria, Belgium, Luxembourg, UK, Mayotte (5)	(0)	South Korea (1)
2000	Afghanistan, Angola, Nigeria, Tanzania, Pakistan (100)	Brazil, India, Indonesia, South Korea, Turkey (72)	Germany, Italy, Spain, UK, Sweden (26)	Bulgaria (1)	(0)
2050	Angola, Burkina Faso, Mali, Niger, Somalia (12)	India, Pakistan, Nigeria, Kenya, Egypt (86)	USA, Brazil, Turkey, Indonesia, Australia (56)	China, Netherlands, Portugal, Czechia, Finland (10)	Japan, Italy, Germany, South Korea, Spain (35)
2100	(0)	Nigeria, Kenya, Iraq, Senegal, Cameroon (42)	USA, India, Brazil, France, Iran (77)	Portugal, China, Estonia, Mexico, Latvia (40)	Japan, Italy, South Korea,Thailand, Germany (40)

**Fig. I-1.**
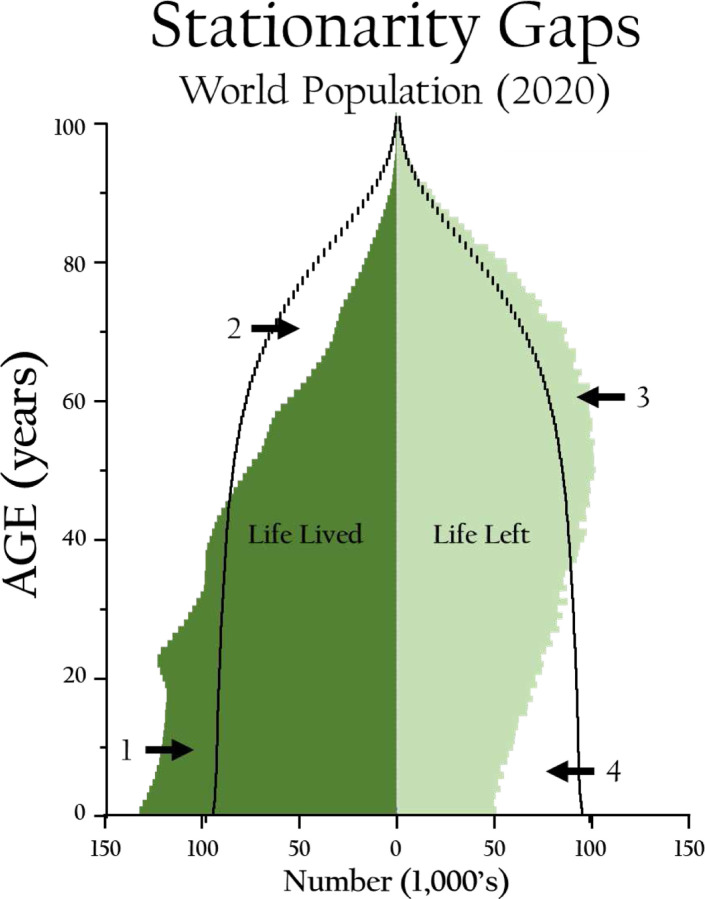
Comparison of the distributions of life lived and life left in the actual 2020 world population (solid asymmetric curve) with those of a stationary population (dashed symmetric curve). In a stationary population, the distributions are identical, producing perfect symmetry. Deviations between the curves represent the Stationary Gap, quantified by the Dissimilarity Index, which measures the extent of reallocation needed to match the distributions. Arrows 1 and 3 indicate excesses in life lived and life left, respectively, while arrows 2 and 4 indicate corresponding deficits relative to the stationary reference.

**Fig. II-1.**
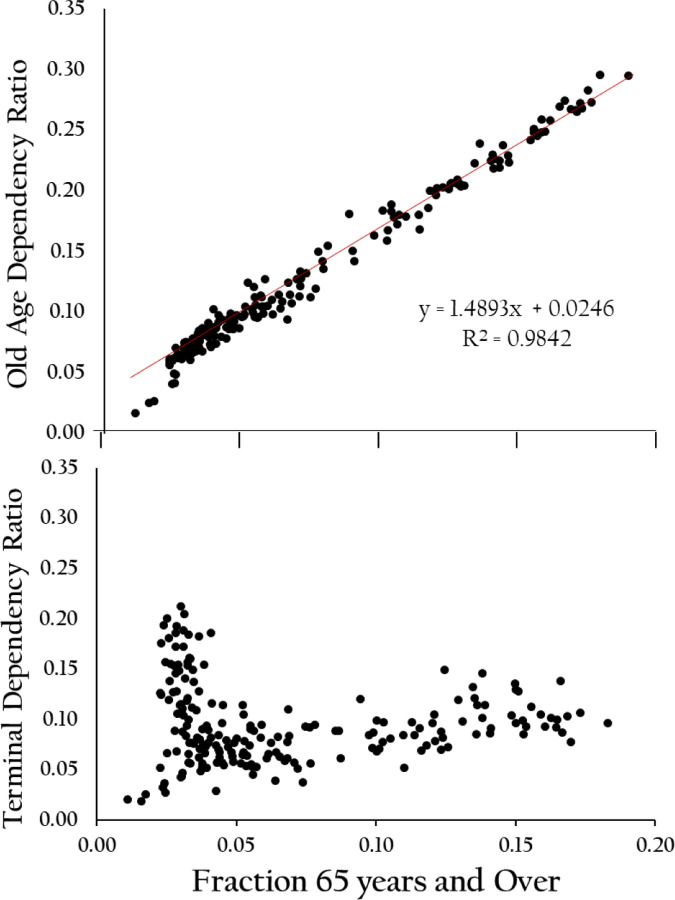
Relationship between the percentage of the population aged 65 and older and two structural aging metrics for 195countries in the year 2000. **(a)** Old-Age Dependency Ratio (OADR) plotted against the fraction of the population aged 65 and over. A strong, near-linear relationship reflects the direct definitional linkage between these two measures, both of which are widely used as conventional indicators of aging burden. **(b)** Terminal Dependency Ratio (TDR) plotted against the same aging proportion. In contrast to the top panel, this relationship shows no strong linear trend, with a wide spread of TDR values at low levels of aging, particularly among countries with under 6% aged 65 and older. This divergence highlights structural mortality variation and suggests that TDR captures a distinct dimension of aging related to proximity to death rather than chronological age.

## Data Availability

Modeling output available at Additional Information this paper. All data used in modeling and analysis are available at:

## Additional Information

### I. Stationarity Gaps: Concept and Visualization”

In demographic analysis, deviations from stationary conditions reveal the structural signatures of population aging. The concept of the **stationary gap** captures the extent to which real-world populations depart from the idealized symmetry of a stationary population, where the distribution of life lived (ages attained) exactly mirrors the distribution of life left (years remaining). In stationary populations, the fraction of individuals at a given age equals the fraction with that number of years remaining, resulting in a perfectly symmetrical demographic profile.

Fig. II-1 illustrates this concept by comparing the actual 2020 world population distribution (solid curve) with the idealized stationary population (dashed curve). The stationary gap is visually evident as the asymmetric divergence between the two curves: excesses in life lived and deficits in life left are marked by directional arrows. The magnitude of this gap is quantified using the Dissimilarity Index (DI), which measures the total proportion of the population that would need to be reallocated across ages to restore symmetry. A larger stationary gap reflects a more structurally aged—and therefore nonstationary—population.

### II. Relationship of fraction 65 and over and Dependency Ratios

Fig. III-1a shows a strong, near-linear relationship between the percentage of the population aged 65 and over and the Old-Age Dependency Ratio (OADR) across 195countries in 2000. The tight clustering around the regression line reflects the definitional link between these two indicators, as both scale directly with the size of the elderly population relative to the working-age population. This close correlation confirms that %≥65 serves as a reliable proxy for OADR in broad demographic classifications. However, the alignment also underscores a key limitation: both metrics capture the same structural dimension of aging and are thus redundant when used together. More importantly, neither provides information about the proximity of elderly individuals to death, the quality or duration of remaining life, or variations in end-of-life burden across countries.

Fig. III-1b presents the relationship between the percentage aged 65 and over and the Terminal Dependency Ratio (TDR), which measures the proportion of the population expected to die within the next five years. Unlike the OADR plot, this figure reveals a distinct bifurcation pattern, particularly among countries where less than 6% of the population is aged 65 or older. In these younger populations, TDR values vary widely—from as low as 0.02 to over 0.20—reflecting stark differences in mortality patterns. High terminal dependency in low-aging countries, especially in Africa and parts of South Asia, indicates that even a small elderly population can face intense end-of-life burden due to high mortality risks shortly after age 65. In contrast, countries with more advanced aging show less variability, suggesting convergence toward more predictable patterns of longevity.

Together, these two panels highlight a critical distinction in the conceptual depth of aging metrics. While %≥65 and OADR are highly correlated and largely interchangeable for classifying aging levels, they offer limited insight into mortality timing or the demographic weight of dying. TDR, by contrast, introduces a second structural dimension that captures the dynamic nature of aging through the lens of mortality proximity. Its variability at low aging levels and convergence at higher ones underscore its potential to differentiate countries within the same aging stage—enabling sub-staging—and to reflect differences in survival trajectories that conventional indicators obscure. These results affirm the value of TDR as a complementary metric that reveals structural aging complexity beyond what traditional measures can detect.

### III. Database

This Excel dataset contains population aging indicators for 195 countries plus the world total, reported for the years 1950, 2000, 2050, and 2100. Each row represents a country-year observation and includes both conventional demographic variables and novel structural aging metrics derived from the binary population framework.

#### Column Descriptions:

A. Number

B. World Region – Geographic region classification of the country.

C. Country – Name of the country or territory.

D. Year – Observation year (1950, 2000, 2050, or 2100).

E. Stage – Successional stage of population aging (1 = Youth Dominance, ..., 5 = Age Dominance), based on the Lifespan Parity Ratio.

F. Population (1,000s) – Total population size, reported in thousands.

G. Birth Rate – Crude birth rate (births per 1,000 population per year).

H. Death Rate – Crude death rate (deaths per 1,000 population per year).

I. Mean Age – Average chronological age of the observed population.

J. Mean Years Remaining – Average number of years left to live across the population (life left).

K. Lifespan Parity Ratio – Ratio of mean years remaining to mean age; values above 1.0 indicate youthful populations, values below 1.0 indicate aged populations.

L. Stationary Population Mean Age – Mean age of the stationary population constructed from the same birth and death rates.

M. Expectation of Life at Birth, e(0) – Standard life expectancy at birth.

N. Stationarity Gap – Dissimilarity index comparing the age distribution and remaining-years distribution; a measure of demographic momentum.

O. Fraction >64 – Proportion of the population aged 65 and older.

P. Old Age Dependency Ratio (15–64) – Ratio of population aged 65+ to the working-age population (15–64).

Q. Terminal Dependency Ratio (15–64) – Ratio of expected deaths within the next 5 years to the working-age population (15–64); a measure of imminent mortality burden.

All demographic metrics are derived from or aligned with United Nations World Population Prospects data and life tables for both sexes combined.
